# Clinical practice guideline on the optimal radiotherapeutic management of brain metastases

**DOI:** 10.1186/1471-2407-5-34

**Published:** 2005-04-04

**Authors:** May N Tsao, Nancy S Lloyd, Rebecca KS Wong, the Supportive Care Guidelines Group of Cancer Care Ontario's Program in Evidence-based Care

**Affiliations:** 1 Department of Radiation Oncology, Toronto-Sunnybrook Regional Cancer Centre, Toronto, Ontario, Canada; 2 Department of Clinical Epidemiology & Biostatistics, McMaster University, Hamilton, Ontario, Canada; 3 Department of Radiation Oncology and the Princess Margaret Hospital, University of Toronto, Toronto, Ontario, Canada

## Abstract

**Background:**

An evidence-based clinical practice guideline on the optimal radiotherapeutic management of single and multiple brain metastases was developed.

**Methods:**

A systematic review and meta-analysis was performed. The Supportive Care Guidelines Group formulated clinical recommendations based on their interpretation of the evidence. External review of the report by Ontario practitioners was obtained through a mailed survey, and final approval was obtained from Cancer Care Ontario's Practice Guidelines Coordinating Committee (PGCC).

**Results:**

One hundred and nine Ontario practitioners responded to the survey (return rate 44%). Ninety-six percent of respondents agreed with the interpretation of the evidence, and 92% agreed that the report should be approved. Minor revisions were made based on feedback from external reviewers and the PGCC. The PGCC approved the final practice guideline report.

**Conclusions:**

For adult patients with a clinical and radiographic diagnosis of brain metastases (single or multiple) we conclude that,

• Surgical excision should be considered for patients with good performance status, minimal or no evidence of extracranial disease, and a surgically accessible single brain metastasis.

• Postoperative whole brain radiotherapy (WBRT) should be considered to reduce the risk of tumour recurrence for patients who have undergone resection of a single brain metastasis.

• Radiosurgery boost with WBRT may improve survival in select patients with unresectable single brain metastases.

• The whole brain should be irradiated for multiple brain metastases. Standard dose-fractionation schedules are 3000 cGy in 10 fractions or 2000 cGy in 5 fractions.

• Radiosensitizers are not recommended outside research studies.

• In select patients, radiosurgery may be considered as boost therapy with WBRT to improve local tumour control. Radiosurgery boost may improve survival in select patients.

• Chemotherapy as primary therapy or chemotherapy with WBRT remains experimental.

• Supportive care is an option but there is a lack of Level 1 evidence as to which subsets of patients should be managed with supportive care alone.

Qualifying statements addressing factors to consider when applying these recommendations are provided in the full report. The rigorous development, external review and approval process has resulted in a practice guideline that is strongly endorsed by Ontario practitioners.

## Background

Brain metastases represent a significant health care problem. An estimated 20–40% of cancer patients will develop multiple brain metastases [1], and 30–40% will develop a single metastasis [2] during the course of their illness. The prognosis for patients is generally poor, and treatment decisions are based on a combination of factors, including survival, quality of life, intracranial progression-free duration, response of brain metastases to treatment, symptom control, neurological function, and toxicity. These outcomes were considered in the systematic review that informed this provincial clinical practice guideline, which was initiated to summarize the evidence and to provide recommendations on the optimal management of brain metastases.

The systematic review and meta-analyses, conducted as the initial step in formulating this practice guideline, are described in a companion document that has been submitted elsewhere for publication [3]. It is also currently undergoing review with the Cochrane Collaboration. Briefly, the evidence was broadly divided into studies aimed at management of a single brain metastasis [4-7] versus those aimed at management of multiple brain metastases [8-33] arising from cancer of any histology. The following interventions were compared in the randomized controlled trials included in the systematic review: for single brain metastasis, whole brain radiotherapy (WBRT) with or without surgery [4-6] and surgery with or without WBRT [7]; for multiple brain metastases, supportive care with or without WBRT [8], various altered dose-fractionation schedules [9-17], WBRT with or without radiosensitizers [18-23], chemotherapy with WBRT [24-28], and WBRT with or without radiosurgery [29-33].

## Methods

### Clinical practice guideline development

This practice guideline was developed by Cancer Care Ontario's Practice Guidelines Initiative (PGI), using the methods of the Practice Guidelines Development Cycle [34]. The practice guideline report is a convenient and up-to-date source of the best available evidence on the role of radiation therapy in adult patients with brain metastases, developed through systematic reviews, evidence synthesis, and input from practitioners in Ontario. The report is intended to promote evidence-based practice. The PGI is editorially independent of Cancer Care Ontario and the Ontario Ministry of Health and Long-term Care. The PGI has a formal standardized process to ensure the currency of each guideline report. This process consists of the periodic review and evaluation of the scientific literature and, where appropriate, integration of this literature with the original guideline information.

Evidence was selected and summarized by two members of the Supportive Care Guidelines Group (SCGG) and methodologists. Members of the SCGG disclosed potential conflict of interest information, reviewed the analysis of the evidence, and prepared draft recommendations. The membership of the SCGG includes palliative care physicians, radiation and medical oncologists, radiation therapists, psychiatrists, nurses, psychologists, an anesthetist, a surgeon, and methodologists. After reviewing the evidence, the SCGG reached consensus on draft recommendations.

External review by Ontario practitioners was obtained through a mailed survey consisting of items that address the quality of the draft practice guideline report and recommendations and whether the recommendations should serve as a practice guideline. The efficacy of the practitioner feedback survey process has been previously described [35]. Final approval of the original guideline report was obtained from the Practice Guidelines Coordinating Committee (PGCC).

### Interpretation of the evidence

#### Single brain metastasis

Two of the three trials using WBRT with or without surgical excision of a single brain metastasis detected an overall survival benefit favouring the addition of surgery. The trial that did not detect a benefit [4], however, included more patients with poorer performance status and a higher proportion of patients with extracranial disease as compared to the other two trials.

The randomized trial by Patchell et al. [7] reported on the use of surgery with or without WBRT. A significant reduction in brain recurrence rates was detected in the surgery and WBRT arm, but there was no significant difference in overall survival.

The methodologic quality of the studies was similar. However, the description of withdrawals and dropouts was variable. Only the Patchell trials [6,7] required magnetic resonance imaging (MRI)-confirmed single metastasis. As such, those trials which relied on brain computed tomography (CT) may have included patients with multiple brain metastases rather than single. The benefit of adding surgery in these patients with truly multiple brain metastases may have been diminished.

In the trials examining the use of surgery and WBRT for single brain metastasis, the WBRT doses were 3000 cGy/10 fractions daily [4], 4000 cGy/20 fractions given twice a day [5], 3600 cGy/12 fractions daily [6] and 5040 cGy/28 fractions daily [7].

The Radiation Therapy Oncology Group (RTOG) trial [31-33] randomized 164 patients to WBRT and radiosurgery boost versus 167 patients to WBRT alone. Overall, there was no improvement in overall survival. An improvement in one-year brain control rates was observed in the radiosurgery boost arm. That trial included a predefined hypothesis to detect a 75% median survival time improvement (80% statistical power) in patients with single brain metastasis. Median survival was 6.5 months in patients with single brain metastasis treated with radiosurgery boost as compared to 4.9 months in patients with single brain metastasis treated with WBRT alone, p = 0.0393.

The evidence provided in the systematic review [3] suggests that surgical resection of a single brain metastasis in a patient with good performance status (Karnofsky Performance Status [KPS] ≥ 70) and stable or no extracranial disease improves overall survival. The addition of WBRT after surgical resection of a single brain metastasis decreases brain recurrence rates. Based on one randomized trial, the use of radiosurgery boost with WBRT was reported to improve survival as compared to WBRT alone in selected patients with single brain metastasis.

#### Multiple brain metastases

One randomized trial [8] examined the use of prednisone with or without WBRT. This was an older trial, with a small sample size of 48 patients, reported in the era prior to CT scanning. The diagnosis of brain metastases was based on outdated criteria; not contemporary CT or MRI criteria. The proportion of patients with improved performance status was similar in the steroid-alone and combined WBRT and steroid arms (63% and 61% respectively). The median survival of the steroid-alone arm was 10 weeks as compared to 14 weeks in the combined arm (p-value not stated). The methodologic quality of that study was poor. Sample size calculations were not described a priori, and a description of dropouts and withdrawals was not provided. Statistical analyses were not performed and therefore, the magnitude of benefit with the use of WBRT over supportive care alone remains unclear, particularly in patients with poor performance status and/or active extracranial disease.

In several randomized controlled trials included in the systematic review [3], a significant benefit in terms of overall survival or symptom control was not detected with altered dose-fractionation schedules as compared with a standard dose-fractionation schedule of 3000 cGy in 10 fractions. The included studies were similar in methodologic quality. Details of randomization (e.g., blinding of randomization) were rarely provided, and complete follow-up was variable among the studies. None of the trials reported on the blinding of outcomes. Furthermore, none of the negative trials commented on confidence intervals or power calculations. A lack of sufficient high-quality evidence precludes recommendations on which treatment regimen(s) provide the greatest improvement in symptom control.

In an attempt to improve the response of brain metastases to treatment, radiosensitizers have been added to WBRT. However, none of the five randomized trials [18-21,23] detected a significant benefit in overall survival or brain metastases response (CR + PR). None of the trials examining the use of radiosensitizers were double-blind. However, the events review committee (ERC) in the gadolinium trial [22,23] was blinded to treatment assignment and reviewed baseline and follow-up data. Based on subgroup analysis, there was a suggestion that recursive partitioning analysis (RPA) Class II lung cancer patients with brain metastases may benefit from the use of motexafin gadolinium and WBRT. This is being further studied in a phase III trial where patients with metastatic non-small cell lung cancer are randomized to WBRT with or without motexafin gadolinium.

In a non-blinded study, Ushio [24] randomized patients with metastatic lung cancer to the brain to one of three groups (WBRT alone, WBRT + chloroethylnitrosoureas, or WBRT + chloroethylnitrosoureas + tegafur). No significant difference in overall survival was seen among the three groups. Brain response rates were significantly different between the WBRT-alone arm and the WBRT + chloroethylnitrosoureas + tegafur arm. However, 12 patients were excluded from the evaluation due to protocol violations, which may have skewed the results of the study given the small number of patients. Two patients died of probable side effects of chemotherapy.

For metastatic small-cell lung cancer, Postmus [25] found no difference in overall survival in patients treated with teniposide alone versus teniposide and WBRT. Although the combined arm had higher brain response rates, there is no comparison with WBRT alone. That study showed that chemotherapy alone is inferior to the use of WBRT and chemotherapy for improved brain metastases response rates. However, it does not address the question as to whether WBRT alone is superior or equivalent to WBRT and chemotherapy for brain response and neuropsychological outcomes.

For metastatic non-small cell lung cancer, Robinet [26] found no difference in overall survival with early versus delayed WBRT when given with chemotherapy. Delayed WBRT was given to intracranial non-responders to chemotherapy. That non-blinded study was powered to detect a 25% improvement in the six-month survival rate. Approximately 13% of patients were inevaluable for intracranial or extracranial response. However, withdrawals and drop-outs were described in terms of numbers and reasons per group. There was a 21% overall response (CR + PR) after two cycles of chemotherapy alone and 20% overall response to chemotherapy and early WBRT. Six-month survival was no different between the two arms. The results confirmed that chemotherapy alone may reduce the size of brain metastases from metastatic non-small cell lung cancer. The timing of WBRT in relation to chemotherapy did not affect survival. However, it was not possible to establish the optimal timing of WBRT when given concurrently with chemotherapy from the results of the Robinet trial [26].

Mornex [27] found no difference in cerebral response rates between combined fotemustine and WBRT versus fotemustine alone in patients with metastatic melanoma to brain. However, there was a significant difference in favour of the combined arm for time to cerebral progression. The most severe side effect was myelosuppression. Delayed grade 3–4 neutropenia occurred in 46% of patients in the fotemustine alone arm and 35% in the combined arm. Delayed grade 3–4 thrombocytopenia occurred in 44% of patients in the fotemustine-alone arm and 38% in the combined arm. That trial did not address the question of whether WBRT alone is superior or equivalent to WBRT and fotemustine in terms of therapeutic benefit and toxicity in patients with metastatic melanoma to the brain.

Antonadou [28] found no difference in overall survival for patients treated with WBRT and temozolamide chemotherapy versus WBRT alone. However, an improved brain response rate was seen in the combined arm. Those results were published in abstract form. Further trials are needed to confirm a benefit in brain control with the addition of chemotherapy to WBRT.

Three trials [29,30,33] reported on the use of radiosurgery in addition to WBRT. Only one of those trials found a benefit to the use of WBRT in addition to radiosurgery for selected patients with 2–4 brain metastases; however, the trial was small (n = 27 patients), and the results were reported early at 60% accrual. The rate of local brain failure was 100% after WBRT and 8% in those treated with boost radiosurgery. Furthermore, the 100% recurrence rate in the WBRT arm was unusually high. There was no significant difference in overall survival, 7.5 months for WBRT and 11 months for patients in the WBRT and boost radiosurgery arm, p = 0.22. As previously mentioned, the RTOG trial [31-33] randomized 164 patients to WBRT and radiosurgery boost versus 167 patients to WBRT alone. No improvement in overall survival was detected. In patients with single brain metastasis treated with radiosurgery boost median survival was 6.5 months as compared to 4.9 months in patients with single brain metastasis treated with WBRT alone, p = 0.0393. Another trial published in abstract form [30] examined the use of Gamma knife radiosurgery (GK RS), WBRT, or both in the treatment of 1–3 brain metastases. There was no difference in overall survival. Local control rates were superior for the GK RS and GK RS + WBRT arms. Subgroup analysis for patients with single brain metastasis in this latter study [30] was not reported. Thus, the use of radiosurgery appears to improve 1-year local control of brain metastases when used in conjunction with WBRT in selected patients. There is Level 1 evidence (three trials) that overall survival is not improved with the addition of radiosurgery boost to WBRT as compared to WBRT. The optimal timing of radiosurgery has not been elucidated. The question of whether radiosurgery should be used as a boost treatment with WBRT, at the time of relapse after WBRT, or used alone, reserving WBRT for future extensive brain relapse, remains unanswered.

### Supportive Care Guidelines Group consensus

Originally proposed as a guideline topic for Cancer Care Ontario's Neuro-Oncology Disease Site Group (NDSG), in 2002 it was decided that the guideline would be developed under the auspices of the SCGG since the view was to maintain a palliative focus. A separate practice guideline on the management of single brain metastases was developed by the NDSG and is consistent with the current guideline. Both the SCGG and NDSG reviewed all draft versions of the guideline. Modifications were made at various stages as per the groups' feedback and the final version was approved in February 2004.

## Results

### Draft recommendations

Based on the evidence described above, the SCGG, with the opinions of the NDSG, formulated the following *draft *recommendations, which were subsequently sent out for external review:

#### Target population

These recommendations apply to adult patients with a clinical and radiographic diagnosis of brain metastases (single or multiple) arising from cancer of any histology.

##### Radiotherapy and surgery for single brain metastasis

• Surgical excision is recommended, in addition to WBRT, for patients with good performance status, minimal or no evidence of extracranial disease, and a surgically accessible single brain metastasis (single or multiple) arising from cancer of any histology.

• Postoperative WBRT should be used to improve brain control for patients who have undergone resection of a single brain metastasis.

##### Radiotherapy for multiple brain metastases

• Whole brain radiotherapy is the recommended volume of treatment for multiple brain metastases. Commonly used dose-fractionation schedules are 3000 cGy in 10 fractions or 2000 cGy in 5 fractions.

• There are no advantages of other altered dose-fractionation WBRT schedules in terms of overall survival or neurologic function.

• The use of radiosensitizers is not recommended outside research studies.

• The optimal use of radiosurgery in the treatment of brain metastases remains to be defined. In patients with one to three brain metastases (less than 3 cm in size) and limited or controlled extracranial disease, radiosurgery may be considered to improve local control either as boost therapy with WBRT or at the time of relapse after WBRT failure.

##### Chemotherapy and whole brain radiotherapy

• The use of chemotherapy as primary therapy for brain metastases (with WBRT used for intracranial non-responders) or the use of chemotherapy with WBRT to treat brain metastases remains experimental.

##### Supportive care and whole brain radiotherapy

• Supportive care alone without WBRT is an option for patients with poor performance status or widely disseminated progressive cancer.

#### Qualifying statements

• The number of patients included in the two trials comparing 3000 cGy in 10 fractions versus 2000 cGy in 5 fractions for multiple brain metastases was small.

• In the trials examining the use of surgery and WBRT for single brain metastasis, the WBRT doses were 3000 cGy/10 fractions daily, 4000 cGy/20 fractions given twice daily, 3600 cGy/12 fractions daily, and 5040 cGy/28 fractions daily. As such, the use of 2000 cGy/5 fractions of WBRT has not been studied directly in this scenario.

### External review process – Ontario practitioner feedback

Feedback on the draft practice guideline report was obtained through a mailed survey of 246 practitioners in Ontario (26 neurosurgeons, 137 medical oncologists, and 83 radiation oncologists). The survey consisted of items evaluating the methods, results, and interpretation of the evidence and whether the draft recommendations should be approved as a practice guideline. Written comments were invited. The SCGG reviewed the results of the survey.

### Results of practitioner feedback

One hundred nine responses were received out of the 246 surveys sent (44% response rate). A summary of the results is provided in Table 1. Of the practitioners who responded, 85 indicated that the report was relevant to their clinical practice and completed the survey. The survey results indicated that 96% of respondents agreed with the interpretation of the evidence and 94% agreed with the draft recommendations as stated. Ninety-two percent of respondents agreed that the report should be approved as a practice guideline. Twenty-three respondents (27%) also provided written comments. The final guideline recommendations, which appear at the end of this report, were modified in accordance with the suggestions from the external reviewers and were subsequently approved by Cancer Care Ontario's Practice Guidelines Coordinating Committee.

### Practice Guidelines Coordinating Committee approval process

The practice guideline report was circulated to the PGCC for review and approval. Four of eight members of the PGCC completed and returned ballots. Three of these members approved the practice guideline report as written, while one member approved the guideline and provided a suggestion for consideration by the SCGG. The suggestion was to revise the wording of the recommendation for single brain metastasis to "considered" rather than "recommended" as the evidence for benefit is not compelling. The SCGG agreed with the suggestion and modified the guideline accordingly.

## Conclusions

### Guideline recommendations

For adult patients with a clinical and radiographic diagnosis of brain metastases (single or multiple) arising from cancer of any histology (except for choriocarcinoma and other germ cell tumours, and hematologic malignancies), we recommend that:

#### Radiotherapy and surgery for single brain metastasis

• Surgical excision should be considered for patients with good performance status, minimal or no evidence of extracranial disease, and a surgically accessible single brain metastasis amenable to complete excision.

• Postoperative WBRT should be considered to reduce the risk of tumour recurrence for patients who have undergone resection of a single brain metastasis.

• Radiosurgery boost with WBRT may also improve survival in select patients with unresectable single brain metastases.

#### Radiotherapy for multiple brain metastases

• The whole brain should be irradiated for multiple brain metastases. Commonly used standard dose-fractionation schedules are 3000 cGy in 10 fractions or 2000 cGy in 5 fractions.

• Altered dose-fractionation WBRT schedules have not demonstrated any advantages in terms of overall survival or neurologic function relative to more commonly used fractionation schedules.

• The use of radiosensitizers is not recommended outside research studies.

• In select patients with up to four brain metastases (up to 4 cm in size) and limited or controlled extracranial disease, radiosurgery may be considered as a boost therapy with WBRT to improve local tumour control. Radiosurgery boost may also improve survival in select patients with unresectable single brain metastases.

#### Chemotherapy and whole brain radiotherapy

• The use of chemotherapy as the primary therapy for brain metastases (with WBRT used for those whose intracranial metastases fail to respond) or the use of chemotherapy with WBRT to treat brain metastases remains experimental.

#### Supportive care and whole brain radiotherapy

• Supportive care alone without WBRT is an option (for example, in patients with poor performance status and progressive extracranial disease). However, there is a lack of Level 1 evidence to guide practitioners as to which subsets of patients with brain metastases should be managed with supportive care alone without WBRT.

To support the application of these recommendations in clinical practice, the following qualifying statements should be considered:

The number of patients included in the two trials comparing 3000 cGy in 10 fractions versus 2000 cGy in 5 fractions for multiple brain metastases was small. In the trials examining the use of surgery and WBRT for single brain metastasis, the WBRT doses were 3000 cGy in 10 fractions daily, 4000 cGy in 20 fractions given twice daily, 3600 cGy in 12 fractions daily, and 5040 cGy in 28 fractions daily. As such, the use of 2000 cGy in 5 fractions of WBRT has not been studied directly in this scenario. The results of the studies may not be generalizable to all tumour types. The majority of the patients in the studies (except the chemotherapy studies) had lung, breast, or colorectal cancer primaries.

## List of abbreviations used

cGy, centigray(s); cm, centimeter(s); CR, complete response; CT, computed tomography; ERC, events review committee; GK RS, gamma knife radiosurgery; Gy, gray(s); KPS, Karnofsky performance status; met, metastasis(es); MRI, magnetic resonance imaging; NDSG, Neuro-Oncology disease site group; PGCC, Practice Guidelines Coordinating Committee; PGI, Practice Guidelines Initiative; PR, partial response; RPA, recursive partitioning analysis; RTOG, Radiation Therapy Oncology Group; SCGG, Supportive Care Guidelines Group; WBRT, whole brain radiotherapy.

## Competing interests

The author(s) declare that they have no competing interests.

## Authors' contributions

MT was the lead author responsible for designing and conducting the systematic review of the literature and the meta-analyses that informed the practice guideline, and for drafting and modifying the practice guideline report. MT is a member of the Supportive Care Guidelines Group and a Radiation Oncologist at the Toronto-Sunnybrook Regional Cancer Centre. NL conducted literature searches and drafted and edited the guideline report during its various stages of development. NL conducted duplicate data extraction and meta-analyses and coordinated input from members of the SCGG. NL updated the literature search, incorporated new data, conducted the practitioner feedback survey, and coordinated approval of the guideline by the PGCC. RW reviewed all drafts of the guideline report and made major contributions to performing the meta-analyses that informed the practice guideline, and provided extensive input to the guideline as a radiation oncologist and methodologist. RW is co-Chair of the Supportive Care Guidelines Group. Members of the SCGG provided feedback on all draft guideline reports.

## Pre-publication history

The pre-publication history for this paper can be accessed here:



## Figures and Tables

**Table 1:** Practitioner responses to eight items on the practitioner feedback survey.

**Item**	**Number (%)**		
	
	**Strongly agree or agree**	**Neither agree nor disagree**	**Strongly disagree or disagree**
The rationale for developing a clinical practice guideline, as stated in the "Choice of Topic" section of the report, is clear.	82 (98)	1 (1)	1 (1)
There is a need for a clinical practice guideline on this topic.	70 (83)	12 (14)	2 (2)
The literature search is relevant and complete.	77 (94)	5 (6)	0
The results of the trials described in the report are interpreted according to my understanding of the data.	81 (96)	3 (4)	0
The draft recommendations in this report are clear.	81 (96)	1 (1)	2 (2)
I agree with the draft recommendations as stated.	79 (94)	2 (2)	3 (4)
This report should be approved as a practice guideline.	77 (92)	6 (7)	1 (1)
If this report were to become a practice guideline, how likely would you be to make use of it in your own practice?	**Very likely or likely**	**Unsure**	**Not at all likely or unlikely**
	
	57 (68)	6 (7)	21 (25)
